# Profile of Hashimoto's Thyroiditis in Sri Lankans: Is There an Increased Risk of Ancillary Pathologies in Hashimoto's Thyroiditis?

**DOI:** 10.4061/2010/124264

**Published:** 2010-10-10

**Authors:** Eranga Himalee Siriweera, Neelakanthi Vajira Illangakoon Ratnatunga

**Affiliations:** Department of Pathology, Faculty of Medicine, University of Peradeniya, Peradeniya 20400, Sri Lanka

## Abstract

Hashimoto's thyroiditis has been reported to be associated with many neoplastic and nonneoplastic thyroid pathologies. This retrospective study aims to determine the demographic profile of Hashimoto's thyroiditis in Sri Lankans, document ancillary pathologies in Hashimoto's thyroiditis, and determine whether there is an increased risk of occurrence of malignancies, benign neoplasms, and nonneoplastic benign lesions in Hashimoto's thyroiditis by comparing with thyroids showing multinodular goiters, follicular adenomas, and colloid nodules. The mean age of Hashimoto's thyroiditis is 43.3 years with the majority in the 41 to 60 year age group and a female to male ratio of 10.3 : 1. This study revealed a statistically significant increase of thyroid malignancies in association with Hashimoto's thyroiditis. The association of Papillary carcinoma, Non-Hodgkin's lymphoma, and Hurthle cell adenoma with Hashimoto's thyroiditis was statistically significant.

## 1. Introduction

Hashimoto's thyroiditis, an autoimmune disease of the thyroid gland is commonly encountered in females of middle age and affects approximately 5% of the population [1–3]. The incidence of Hashimoto's thyroiditis in the west is approximately 0.3–1.5 cases per 1,000 population per year [4]. A study conducted in Malaysia has shown interesting ethnic differences in Hashimoto's thyroiditis such as high prevalence among Indians when compared with Chinese and Malay nationals [5].

Hashimoto's thyroiditis is defined histologically by the presence of diffuse lymphocytic infiltrates, lymphoid follicles with reactive germinal centers, Hurthle cell change of the follicular epithelial cells, parenchymal atrophy, and fibrosis. Hashimoto's thyroiditis has been reported to be associated with many neoplastic and nonneoplastic thyroid pathologies. The rate of occurrence of thyroid malignancies in patients with Hashimoto's thyroiditis ranges from 0.5% to 14% [1, 6]. The more commonly reported malignancies are B- and T-cell Non-Hodgkin's lymphoma and Papillary carcinoma of the thyroid [2, 7]. Other neoplasms such as follicular carcinoma [2], anaplastic thyroid carcinoma [2], medullary carcinoma [2, 6], Hodgkin's lymphoma [7], mucoepidermoid thyroid carcinoma [8], and primary squamous cell carcinoma [9] have also been reported. A review of the literature revealed coexistence with many benign lesions such as intrathyroidal lymphoepithelial cysts [10, 11], Riedel's thyroiditis [12], benign thyroid nodules, and goiter [13] and plasma cell granuloma [14].

There is long-standing controversy regarding the association between Hashimoto's thyroiditis and follicular cell-derived thyroid tumours. This association was first proposed more than 50 years ago by Dailey et al. who found a significantly higher frequency of thyroid carcinomas and adenomas in surgically removed glands with Hashimoto's thyroiditis than in glands unaffected by this disease [15]. Most of the malignancies found in their series were papillary carcinomas. The subsequent studies yielded conflicting results, reporting either very high or low incidence of thyroid carcinoma in association with Hashimoto's thyroiditis [16–18]. 

The objective of this study is to determine the demographic profile of Hashimoto's thyroiditis in Sri Lankans, detect the ancillary pathologies associated with Hashimoto's thyroiditis, and to determine whether there is an increased risk of occurrence of malignancies, benign neoplasms, and nonneoplastic benign lesions in Hashimoto's thyroiditis.

## 2. Materials and Methods

A retrospective review of 5357 consecutive histopathology records of total and subtotal thyroidectomy specimens received over a fifteen year period formed the basis of this study. Data on demographic profile (age and gender), presence or absence of Hashimoto's thyroiditis, and the type of ancillary pathology were gathered. No clinical data were available. A total of 349 Hashimoto's thyroiditis cases were collected. 2663 multinodular goiters, 1166 follicular adenomas, and 631 colloid nodules were gathered for comparison with Hashimoto's thyroiditis. All categories were stratified according to age groups of 0–20 years, 21–40 years, 41–60 years, and 61 or more than 61 years. Ancillary pathologies seen in each of these categories were recorded in these age groups. The gender and age distribution of Hashimoto's thyroiditis was analysed. The percentages of the ancillary pathologies seen in the specified age groups were calculated for all the categories. To determine whether there is an increased occurrence of ancillary pathologies in a background of Hashimoto's thyroiditis age-matched groups of Hashimoto's thyroiditis were comparatively analyzed with thyroids affected by multinodular goiters, follicular adenomas, and colloid nodules. The statistical analysis of the data was carried out using the Minitab version 13.20 software. When the sample size was too small Fishers exact test (FET) was performed. The statistical associations between categories were calculated at a level of significance (*α*) of 0.05.

## 3. Results

The incidence of Hashimoto's thyroiditis in this study of 5357 cases is 6.51%, which is similar to the incidence (6.6%) reported by Chesky as quoted by Carson et al. [19]. Of the 349 cases 318 (91.12%) were females and 31 (8.88%) were males indicating a female to male ratio of 10.3 : 1. The age ranged from 8 to 89 years with a mean age of 43.3 years. The majority of the cases were seen in the 41–60 year age group (53.3%) followed by 21–40 years (34.96%), 61 years and above (6.02%) and 0–20 years (3.44%). A female predominance was seen in all the age groups. In the 0–20 year age group only females were affected by Hashimoto's thyroiditis. In all the other age groups more than 85% comprised of females (Table 1).

The ancillary pathologies found in association with Hashimotos' thyroiditis were papillary carcinoma (PC), follicular carcinoma (FC), medullary carcinoma (MC), anaplastic carcinoma (AnC), Non-Hodgkin's lymphoma (NHL), follicular adenoma (FA), Hurthle cell adenoma (HA), multinodular goiter (MNG), colloid goiter (CG), colloid nodule (CN), hyperplastic nodule (HypN), and benign cysts (Cyst). In our study 142 cases (40.69%) displayed an ancillary pathology. Of the 349 cases an associated malignancy was seen in 46 cases (13.18%). Of the malignancies 72% were papillary carcinomas, followed by follicular carcinoma (17%), Non-Hodgkin's lymphoma (6%), medullary carcinoma (2%), and anaplastic carcinoma (2%). As such the commonest epithelial malignancy seen in Hashimoto's thyroiditis was papillary carcinoma. An associated benign neoplasm was detected in 54 cases (15.47%). Of this 66.67% were follicular adenomas and 33.33% were Hurthle cell adenomas. In 67 cases (19.2%) an ancillary benign lesion was seen. This included multinodular goiters (52.24%), colloid nodule (34.33%), hyperplastic nodule (7.46%), benign cysts (4.48%), and colloid goiter (1.5%) ( Table 2).

Of the 142 cases in which an ancillary pathology was detected 17.6% showed multiple pathologies. Papillary carcinoma was seen as a dual pathology with Hurthle cell adenoma (2.1%), multinodular goiter (2.1%), follicular carcinoma (1.4%), follicular adenoma (0.7%), and anaplastic carcinoma (0.7%). Hurthle cell adenoma was seen simultaneously with follicular carcinoma (0.7%), multinodular goiter (0.7%) and follicular adenoma (0.7%). The other dual pathologies detected were, follicular adenoma with colloid nodule (0.7%) and multinodular goiter (1.4%) and multinodular goiter with colloid nodule (0.7%) and hyperplastic nodule (1.4%). Several other multiple pathologies were also identified. Papillary carcinoma and Hurthle cell adenoma was seen with follicular adenoma (0.7%), colloid nodule (0.7%), and follicular carcinoma (0.7%). Papillary carcinoma with follicular adenoma and multinodular goiter (0.7%) and multinodular goiter and colloid nodule with follicular adenoma (0.7%) and hyperplastic nodule (0.7%) were observed.

With reference to gender distribution of the ancillary pathologies females showed a higher percentage of malignancies (91.3%), benign neoplasms (75.93%) as well as benign nonneoplastic lesions (85.07%) than males which were statistically significant (*P* < .001). When the ancillary pathologies were analyzed separately all ancillary pathologies except Hurthle cell adenoma and anaplastic carcinoma occurred in higher percentages in females. Medullary carcinoma, multinodular goiter, colloid goiter, and benign cysts were seen exclusively in females. 96.97% of papillary carcinomas, 91.67% of follicular adenomas, 87.5% of follicular carcinomas, 66.67% of Non-Hodgkin's lymphomas, 65.22% of colloid nodules and 60% of hyperplastic nodules, were detected in females. The two instances where a male predominance was seen were Hurthle cell adenoma (57.90%) and anaplastic carcinoma (100%).

When considering the distribution of ancillary pathologies in the specified age groups it was evident that the only malignancy detected in the 0–20 years and 61 years and above was papillary carcinoma. The frequency of papillary carcinoma in the specified age groups varied between 8.33% (0–20 years) and 10.23% (41–60 years). Follicular carcinomas have occurred between 21–60 years of age and all Non-Hodgkin's lymphomas were seen in the 21–40 year age group. A single case each of anaplastic carcinoma and medullary carcinoma was seen in the 41–60 years and 21–40 years age group respectively. Of the benign neoplasms Hurthle cell adenoma was seen between 21–60 years and follicular adenoma was seen in patients above 20 years. Of the nonneoplastic benign lesions multinodular goiters and colloid nodules were seen in association with Hashimoto's thyroiditis in all the age groups. Colloid goiter, hyperplastic nodule, and benign cysts which included a branchial cyst were seen in the 41–60 year age group. 

The comparison of the percentages of ancillary pathologies seen in association with Hashimoto's thyroiditis and thyroids affected by multinodular goiters, follicular adenomas, and colloid nodules is shown in Table 3.

Papillary carcinoma, medullary carcinoma, anaplastic carcinoma, Non-Hodgkin's lymphoma, Hurthle cell adenoma, and benign cysts were seen in higher percentages in association with Hashimoto's thyroiditis when compared with thyroids affected by other pathologies (Table 3). A statistically significant increase in occurrence of papillary carcinoma, Non-Hodgkin's lymphoma, and Hurthle cell adenoma were seen in Hashimoto's thyroiditis when compared with thyroids affected by multinodular goiters, follicular adenomas, and colloid nodules. A statistically significant association of colloid nodule with Hashimoto's thyroiditis was seen when compared with multinodular goiters.

The ancillary pathologies in Hashimoto's thyroiditis were then compared with age-matched groups of thyroids affected by multinodular goiter, follicular adenoma, and colloid nodule. A statistically significant increase of papillary carcinoma in the 21–40 year (*P* value = .008) and 41–60 year (*P* value <.001) age group, Non-Hodgkin's lymphoma (FET *P* value = .001) in the 21–40 year age group, Hurthle cell adenoma in the age groups 21–40 years (*P* value = .008), and 41–60 years (*P* value = .003) and follicular adenoma in >61 year age group (*P* value = .02) were noted in association with Hashimoto's thyroiditis when compared with multinodular goiters. When Hashimoto's thyroiditis and follicular adenomas were comparatively analyzed papillary carcinoma in the 21–40 year (FET *P* value = .019) and 41–60 year (FET *P* value = .007) age groups, Non-Hodgkin's lymphoma (FET *P* value = .004) in the 21–40 year age group, Hurthle cell adenoma in the age groups 21–40 years (FET *P* value <.001), and 41–60 years (FET *P* value <.001) occurred in significantly higher frequencies in Hashimoto's thyroiditis. Comparative analysis between Hashimoto's thyroiditis and colloid nodules revealed a significantly increased occurrence of papillary carcinoma (FET *P* value = .016) in the 21–40 year age group, Non-Hodgkin's lymphoma (FET *P* value = .033) in the 21–40 year age group, Hurthle cell adenoma in the age groups 21–40 years (FET *P* value <.001) and 41–60 years (FET *P* value <.012) in association of Hashimoto's thyroiditis.

## 4. Discussion

The gender distribution of Hashimoto's thyroiditis revealed a female predominance with a female to male ratio of 10.3 : 1 which is similar to the reported female to male ratio that ranges from 4 : 1 to 15 : 1 [2, 3]. Most cases of Hashimoto's thyroiditis were detected in the 5th and 6th decades of life with 53.3% of the cases occurring in this age group followed by 3rd to 4th decades (35%). These findings are similar to those of other studies conducted in the west in which Hashimoto's thyroiditis is reported to be diagnosed between 4th to 6th decades of life [2]. 

According to the present study malignancies as well as benign neoplasms coexisted with Hashimoto's thyroiditis. Papillary carcinomas have occurred in all the age groups, whereas follicular carcinoma, medullary carcinoma, anaplastic carcinoma, Non-Hodgkin's lymphoma, Hurthle cell adenoma and follicular adenoma, were seen in patients over 20 years of age. Females appeared to be at a greater risk of having an ancillary malignant or benign lesion in a background of Hashimoto's thyroiditis. Of note is the higher percentage of Hurthle cell adenomas and anaplastic carcinomas in males.

In our study of 349 cases with Hashimoto's thyroiditis, 40.69% had one or more associated pathologies. Of this 17.6% showed two or more pathologies associated with Hashimoto's thyroiditis. Papillary carcinoma was seen in combination with Hurthle cell adenoma, multinodular goiter, follicular carcinoma, follicular adenoma, and anaplastic carcinoma. Hurthle cell adenoma was seen simultaneously with follicular carcinoma, multinodular goiter, and follicular adenoma. The other concurrent pathologies detected were follicular adenoma with colloid nodule and multinodular goiter and multinodular goiter with colloid nodule and hyperplastic nodule. 

Since the initial description by Marjolin in 1828, chronic inflammation has been increasingly recognized to play a major role in development of cancer. However the association between Hashimoto's thyroiditis and thyroid malignancies remain controversial [2]. We observed that in 349 patients with Hashimoto's thyroiditis 13.18% had coexistent malignancies. The malignancies detected were papillary carcinoma (9.46%), follicular carcinoma (2.29%), Non-Hodgkin's lymphoma (0.86%), medullary carcinoma (0.29%), and anaplastic carcinoma (0.29%). The percentages of thyroid malignancies in thyroids affected by multinodular goiter, follicular adenoma and colloid nodule were 3.04%, 5.4% and 8.34%, respectively. The increase of thyroid malignancies in Hashimoto's thyroiditis was statistically significant. There is lack of consensus as to whether there is an increased incidence of thyroid cancer associated with Hashimoto's thyroiditis [2, 15–17, 19]. According to our study the percentage of thyroid epithelial malignancies in Hashimoto's thyroiditis was 12.32% compared with 3% in multinodular goiters, 5.32% in follicular adenomas, and 8.24% in colloid nodules. The higher percentage of thyroid epithelial malignancies in Hashimoto's thyroiditis was statistically significant. The coincidence of Hashimoto's thyroiditis and thyroid carcinoma will be influenced by geography, histologic criteria in diagnosing Hashimoto's thyroiditis, and the diligence of the pathologist in searching for thyroid cancer which may partly explain these differences [16]. When the malignancies were analyzed separately papillary carcinoma between 21–60 years and Non-Hodgkin's lymphoma in the 21–40 year age group showed a statistically significant association with Hashimoto's thyroiditis. The association between Non-Hodgkin's lymphoma and preexisting Hashimoto's thyroiditis has been well recognized which is thought to develop from acquired intrathyroidal lymphoid tissue in the setting of autoimmune thyroiditis [20–22]. The incidence of Hashimoto's thyroiditis in patients with lymphoma of the thyroid is 25% [16].

Of the benign neoplasms a statistically significant increase of Hurthle cell adenoma was seen in association with Hashimoto's thyroiditis. Similar results were reported in a study conducted by Dailey et al. [15]. Patients with Hashimoto's thyroiditis should therefore be observed closely for coexistent neoplasia specially Non-Hodgkin's lymphoma and Hurthle cell adenoma.

Of the ancillary pathologies detected in this study follicular carcinoma, medullary carcinoma, anaplastic carcinoma, follicular adenoma, multinodular goiter, colloid goiter, colloid nodule, hyperplastic nodule, and benign cysts showed no statistically significant association with Hashimoto's thyroiditis. Of note was a branchial cyst in a 59-year-old female with which Hashimoto's thyroiditis is reported to be associated with [10, 11].

The limitations of this study are that this is based on a pathological series and clinical details are not available. As the study is based on surgically removed thyroid glands this could have influenced the results. A study based on patients with a clinical diagnosis of Hashimoto's thyroiditis would probably address this issue, avoiding the limitations of a surgical bias.

Detection of ancillary pathologies in Hashimoto's thyroiditis has diagnostic and prognostic implications. When considering diagnosis, cytology plays an imminent role where serological and imaging investigations are not freely available as in Sri Lanka. The cytological features of Hashimoto's thyroiditis can overlap with features of lesions such as follicular neoplasms, Hurthle cell neoplasm, papillary, and goitrous nodules that can coexist with Hashimoto's thyroiditis [1, 3, 19]. Also cytological features of Hashimoto's thyroiditis may dominate the smears and overshadow the coexisting neoplasm causing false negative results. With reference to prognosis it has been reported that malignant neoplasms associated with Hashimoto's thyroiditis are usually of lower grades of malignancy which relates to longer survival of the patients [6, 15].

## 5. Conclusion

In conclusion this study revealed that Hashimoto's thyroiditis in the studied Sri Lankan population affects patients in the 4th to 6th decade of life with a female predominance. Many malignant and benign neoplasms and other thyroid pathologies coexist with Hashimoto's thyroiditis and are more common in patients over 20 years of age and in females. A statistically significantly higher percentage of papillary carcinoma, Non-Hodgkin's lymphoma and Hurthle cell adenoma were seen in association with Hashimoto's thyroiditis when compared with thyroids displaying other pathologies. 

## Figures and Tables

**Table 1:** Gender distribution of Hashimoto's thyroiditis. (Percentages from a total of 349 Hashimoto's thyroiditis is shown within brackets).

Age group (years)	Females	Males	Total
0–20	12 (3.44%)	0	12
21–40	109 (31.23%)	13 (3.72%)	122
41–60	171 (48.99%)	15 (4.29%)	186
61 and above	18 (5.16%)	3 (0.86%)	21
Not known	8 (2.29%)	0	8

**Table 2:** Number of ancillary pathologies associated with Hashimoto's thyroiditis.

Age(years)	PC	FC	MC	AnC	NHL	FA	HA	MNG	CG	CN	HyN	Cysts
0–20 (12)	1	0	0	0	0	0	0	1	0	2	0	0
21–40 (122)	11	3	1	0	3	11	8	10	0	8	0	1
41–60 (186)	19	4	0	1	0	21	9	20	1	11	5	2
>61 (21)	2	0	0	0	0	4	0	3	0	1	0	0
Not known	0	1	0	0	0	0	1	1	0	1	0	0

Total	33	8	1	1	3	36	18	35	1	23	5	3
%	9.46	2.29	0.29	0.29	0.86	10.32	5.16	10.03	0.29	6.59	1.43	0.86

	46 (13.18%)					54 (15.47%)		67 (19.2%)				

**Table 3:** Comparison of the percentages of ancillary pathologies seen in association with Hashimoto's thyroiditis and thyroids affected by multinodular goiters (MNG), follicular adenomas (FA), and colloid nodules (CN).

	PC	FC	MC	AnC	NHL	FA	HA	MNG	CG	CN	HyN	Cysts
HT	9.46	2.29	0.27	0.27	0.86	10.32	5.16	10.03	0.27	6.59	1.43	0.86
MNG	1.61	1.16	0.08	0.08	0.04	8.26	0.45	—	—	2.93	1.20	0.41
FA	3.17	2.06	0	0	0	—	1.03	18.87	0.26	7.12	1.46	0.43
CN	5.07	3.01	0	0	0	13.15	0.95	12.36	0.63	—	0.79	0.63
